# Graphene Oxide Molecularly Imprinted Polymers as Novel Adsorbents for Solid-Phase Microextraction for Selective Determination of Norfloxacin in the Marine Environment

**DOI:** 10.3390/polym14091839

**Published:** 2022-04-29

**Authors:** Jianlei Chen, Liju Tan, Zhengguo Cui, Keming Qu, Jiangtao Wang

**Affiliations:** 1 Yellow Sea Fisheries Research Institute, Chinese Academy of Fishery Sciences, Qingdao 266071, China; chenjl@ysfri.ac.cn (J.C.); qukm@ysfri.ac.cn (K.Q.); 2 Key Laboratory of Marine Chemistry Theory and Technology, Ministry of Education, Ocean University of China, Qingdao 266100, China; lijutan@ouc.edu.cn

**Keywords:** marine environment, norfloxacin, solid-phase microextraction, graphene oxide, molecularly imprinted polymers

## Abstract

In this study, a novel sample pretreatment strategy of solid-phase microextraction using graphene oxide molecularly imprinted polymers as adsorbents coupled with high-performance liquid chromatography was developed to detect norfloxacin in the marine environment. As a carrier, the imprinted polymers were synthesized by precipitation polymerization with graphene oxide. Compared with graphene oxide non-imprinted polymers, the graphene oxide molecularly imprinted polymers exhibited higher adsorption capacity towards norfloxacin. The synthesized polymeric materials were packed into a molecularly imprinted solid-phase microextraction cartridge, and critical parameters affecting the extraction process were optimized. Under the optimized molecular imprinted solid-phase microextraction condition, the proposed method was applied to the analysis of norfloxacin for seawater and fish with satisfactory recovery (90.1–102.7%) and low relative standard deviation (2.06–5.29%, n = 3). The limit of detection was 0.15 μg L^−1^ and 0.10 μg kg^−1^ for seawater and fish, respectively. The study revealed that the proposed molecularly imprinted solid-phase microextraction represents an attractive sample pretreatment strategy for the analysis of norfloxacin in the marine environment.

## 1. Introduction

Norfloxacin is a typical antibacterial agent of quinolones which can effectively inhibit Gram bacteria and is widely used in the treatment of respiratory tract infections, skin infections, urinary system infections, infectious diarrhea, and other infectious diseases [1,2]. Norfloxacin is widely used in the prevention and treatment of infectious diseases in humans, animal husbandry, and aquaculture because of its low price and strong antibacterial properties [3,4]. However, the abuse of antibiotics has become more and more serious in recent years [5]. The widespread use of norfloxacin has resulted in the accumulation of large amounts of residual antibiotic in biological samples and animal sources [6]; the abundance of these substances has led to the development of drug resistance in receptors and may cause allergic reactions in humans [7,8]. In addition, the residual norfloxacin can also adversely affect the growth of young people, leading to stunted growth, which can endanger life when severe [9].

Traditional detection methods for norfloxacin mainly include enzyme-linked immunosorbent assay [10,11], gas chromatography, and liquid chromatography [12,13], among which high-performance liquid chromatography (HPLC) has higher accuracy and lower detection limits [14,15]. Sample pre-treatment is a critical step in the analytical procedure of target analytes, especially in complex matrices. Conventional sample pretreatment techniques, such as solid-phase extraction and liquid-liquid extraction, have been widely used to extract and preconcentrate norfloxacin in diverse samples [16]. Although liquid-liquid extraction produces clean extracts, some drawbacks have limited its use as a sample concentration method, such as the formation of emulsions, the consumption of large amounts of organic solvents, and limited selectivity. Solid-phase extraction can effectively address some of the defects of liquid-liquid extraction, and some adsorbents it uses can preconcentrate samples well. Lee et al. concentrated norfloxacin by SPE and further assessed it in wastewater samples [17]. Gaballah et al. used a coupled method of online solid-phase extraction with ultra-high performance liquid chromatography to concentrate and assess norfloxacin in swine manure [18]. However, the traditional pretreatment method based on a solid-phase extraction column usually requires a large amount of adsorbent, which increases the cost of sample analysis. Therefore, it is important to develop a simple, rapid, and efficient method for micro-scale sample preparation using fewer adsorbents and organic solvents. A solid-phase microextraction (SPME) method has been proposed for the extraction of norfloxacin [19,20]. Based on the different distribution coefficients of the analyte between the sample matrix and polymer film coating, SPME can concentrate the target analyte on the coating, which can integrate the extraction, concentration, and purification of the target analyte. Yu et al. developed magnetic solid-phase microextraction for the rapid detection of norfloxacin which had high enrichment [21]. Other methods, such as in-tube solid-phase microextraction, hollow-fiber microextraction, immunoaffinity microextraction, and dispersive liquid-liquid microextraction [22,23,24], have also been utilized in the concentration and analysis of norfloxacin.

In addition, considering the complex marine matrix environment, it is of great importance to develop new SPME with selectivity and anti-interference capability for the concentration of norfloxacin in the marine environment. Molecularly imprinted polymers (MIPs) are synthetic polymers that can simulate the selective recognition of specific target components by the “antigen-antibody” recognition mechanism and are especially suitable as the adsorbent for SPME [25,26,27]. Traditional MIPs are simply bulk polymers synthesized by bulk polymerization [28,29], which has certain disadvantages, such as few binding sites, a low rate of mass transfer, deep embedding, and difficult elution of the template, etc. In contrast, the imprinted sites of surface MIPs are located at the surface of the material, which can improve the recognition response and adsorption capacity to imprinted molecules, overcoming the shortcomings of traditional MIPs, and thus improving the sensitivity of detection methods [30,31]. An increasing number of reports have considered the application of carbon-based materials in polymer synthesis and environmental analysis [32,33]. Graphene oxide (GO) is a derivative of graphene, consisting of 2D honeycomb lattice structures with oxygen-containing functional groups, such as hydroxyl, carboxyl, and epoxy groups. The hydroxyl and epoxy groups are mainly located on the surface of GO, while the carboxyl group is located at the edge of GO [34]. The presence of oxygen-containing functional groups makes GO hydrophilic and able more easily to participate in the preparation of imprinted polymers with noncovalent or covalent functionalization methods. In addition, GO–based MIP composite is beneficial to reduce the “embedding” phenomenon of templates and improve the elution efficiency of template molecules [35]. Moreover, by increasing the surface properties of the composite material, the kinetic characteristics of adsorption efficiency can be improved which can reduce the selective adsorption time [36]. Although norfloxacin determination based on MIPs has been reported, these studies only used traditional MIPs and researched biological samples [37]. Few reports have focused on the determination of norfloxacin in marine environmental samples based on MIPs combining both GO and SPME [38,39].

Based on the above reports, this investigation aimed to synthesize MIPs based on GO, using precipitation polymerization combined with SPME for marine environment sample preparation and the determination of norfloxacin by HPLC. The characterization of the polymers was investigated using a scanning electron microscope (SEM) and Fourier transform infrared spectroscopy (FT–IR). Furthermore, the variables in the analysis, such as organic solvent, pH, extraction time, and eluent, were optimized for the molecularly imprinted solid-phase microextraction (MISPME) procedure. Under the optimal conditions, the proposed method was used for the detection of trace norfloxacin from natural seawater and fish samples.

## 2. Experimental Procedure

### 2.1. Materials and Chemicals

Norfloxacin (NOR), ofloxacin, ciprofloxacin, sulfadiazine, sulfamethazine, methacrylic acid (MAA), and ethyleneglycol dimethacrylate (EGDMA) were purchased from the Aladdin Reagent Company (Shanghai, China). Azodiisobutyronitrile (AIBN) and acetic acid were provided by the Kermel Chemical Company (Tianjin, China). Methanol and acetonitrile were HPLC grade and supplied by Merck (Darmstadt, Germany). Scaly graphite, triethylamine, acetone, tetrahydrofuran, hydrogen peroxide (H_2_O_2_), hydrochloric acid (HCl), sodium hydroxide (NaOH), and sodium nitrate (NaNO_3_) were obtained from the Sinopharm Chemical Reagent Co. (Shanghai, China). The water used in the experiment was Milli-Q water prepared using Millipore equipment (Bedford, MA, USA). The stock standard solution of norfloxacin (1000 mg L^−1^) was prepared in the acetonitrile and diluted to different concentrations as working solutions with acetonitrile/water (19/81, *v*/*v*) before use.

### 2.2. HPLC Analysis

The concentrations of the target analyte norfloxacin were determined on a Hitachi L-2000 HPLC system including a Hitachi L-2130 pump, an L-2200 liquid autosampler, an L-2455 diode array detector (DAD), and an L-2300 column compartment. The analytical column was a 4.6 × 250 mm, 5 μm LaChrom C18 column (Hitachi, Tokyo, Japan). Acetonitrile and 0.05 M phosphoric acid aqueous solutions (pH 2.4 conditioned by triethylamine) with a ratio of 19/81 (*v*/*v*) were used as the mobile phase at a flow rate of 1 mL min^−1^ and the sample injection volume was 10 μL. The detection wavelength was 278 nm, and the column temperature was kept at 30 °C.

### 2.3. Preparation of GO

GO was prepared according to an improved Hummers method [40,41]. A quantity of 0.5 g of scaly graphite, 0.5 g of NaNO_3_ and 23 mL of concentrated H_2_SO_4_ were mixed and stirred in the ice bath for 2 h and the temperature kept to less than 4 °C. A quantity of 3 g of KMnO_4_ was added slowly into the reaction solution, stirred for 30 min, and the temperature of the whole process was kept below 10 °C. The temperature was increased to 30–35 °C and the solution stirred for 2 h. A quantity of 80 mL of Milli-Q water was slowly added to the reaction mixture slowly and the mixture was stirred for 30 min at 95 °C. A quantity of 60 mL of Milli-Q water was then slowly added to the mixture. A quantity of 3 mL of H_2_O_2_ was slowly added when the temperature had reduced to 75–80 °C. The obtained precipitate was washed with 5% HCl until no SO_4_^2ȡ^ was detected, and then washed with Milli-Q water until the pH became neutral. Finally, the solid product was dried in a vacuum oven at 40 °C for 48 h to obtain GO.

### 2.4. The Preparation of Polymers

The graphene oxide molecularly imprinted polymers (GO/MIPs) were prepared by precipitation polymerization. A quantity of 15.97 mg of norfloxacin (template) and 170 μL of methacrylic acid (monomer) were dissolved in 45 mL of acetonitrile in a three-necked flask with ultrasonic treatment for 15 min. Then, 5 mL of GO aqueous solution (1 mg mL^−1^) was added to the mixture and stirred well with a magnetic stirrer. A quantity of 1.88 mL of ethyleneglycol dimethacrylate and 40 mg of azodiisobutyronitrile were added to the solution, and the above mixture was thermally initiated for 24 h in an N_2_ environment under 60 °C in a water bath. The polymers were washed with methanol and water successively more than three times to remove the residual solvent. The obtained particles were eluted using Soxhlet extraction with methanol/acetic acid (90/10, *v/v*) as the solution used to remove the templates until no norfloxacin could be detected. Finally, the GO/MIPs were washed with methanol and water to remove the residual acetic acid and dried in a vacuum oven at 40 °C for 24 h. The graphene oxide non-imprinted polymers (GO/NIPs) were prepared by the same process as GO/MIPs except without the addition of the template in the polymerization process.

### 2.5. Characterization of the Polymers

The surface morphology of the polymers was examined by S-4800 cold field emission scanning electron microscopy (SEM, Hitachi, Japan) and the characteristic functional groups of GO/MIPs and GO/NIPs were confirmed by analyzing Fourier transform infrared (FT-IR) spectra with an AVATAR360 FTIR spectrophotometer (Nicolet Instrument Corporation, Madison, WI, USA). The Brunauer–Emmett–Teller (BET) and pore size distribution of the obtained polymers were calculated based on the nitrogen adsorption-desorption results using an ASAP 2020plus HD88 adsorption analyzer (Micromeritics, Norcross, GA, USA).

### 2.6. Adsorption Experiments

The adsorption performance of GO/NIPs and GO/MIPs to norfloxacin was investigated through dynamic and static adsorption experiments [42]. The mass transfer rate between the polymer and the analyte was studied by kinetic experiment. A quantity of 10 mg of GO/MIPs (or GO/NIPs) was added to 1 mL of norfloxacin standard solution (10 mg L^−1^). After being shaken for different times (0–180 min) at room temperature, the mixed solution was passed through a 0.22 μm filter membrane and the remaining norfloxacin in the filtrate were detected by HPLC–DAD. The adsorption amounts of the polymers to NOR at different times were calculated using the following equation:

$$Q_{t}=\frac{(C_{0}-C_{t})\times V}{m}$$

where Q_t_ (μg g^−1^) is the adsorption capacity of the polymer to NOR at different times, C_0_ (mg L^−1^) is the initial standard solution concentration of norfloxacin, C_t_ (mg L^−1^) is norfloxacin concentration in the solution at time t, V (mL) is the volume of the norfloxacin standard solution, and m (g) is the mass of the added polymers.

The static adsorption experiment was carried out by making GO/NIPs or GO/MIPs reach adsorption equilibrium with different known concentrations of norfloxacin standard solutions. A quantity of 5 mg of GO/MIPs (or GO/NIPs) was added to 1 mL of norfloxacin standard solutions (0–200 mg L^−1^). After being shaken for 4 h at room temperature, the mixture was passed through a 0.22 μm filter membrane and the NOR in the filtrate was determined by HPLC. The adsorption capacity of the polymers to norfloxacin in the standard solution with different concentrations was calculated using the following equation.

$$Q_{e}=\frac{(C_{0}-C_{e})\times V}{m}$$

where Q_e_ (mg g^−1^) is the binding capacity of polymers to NOR, C_0_ and C_e_ (mg L^−1^) are the initial and final concentrations of norfloxacin in the solution, respectively, V (mL) is the volume of the norfloxacin standard solution, and m (g) is the mass of the added polymers.

### 2.7. Preparation of MISPME Procedure

**Scheme 1:**
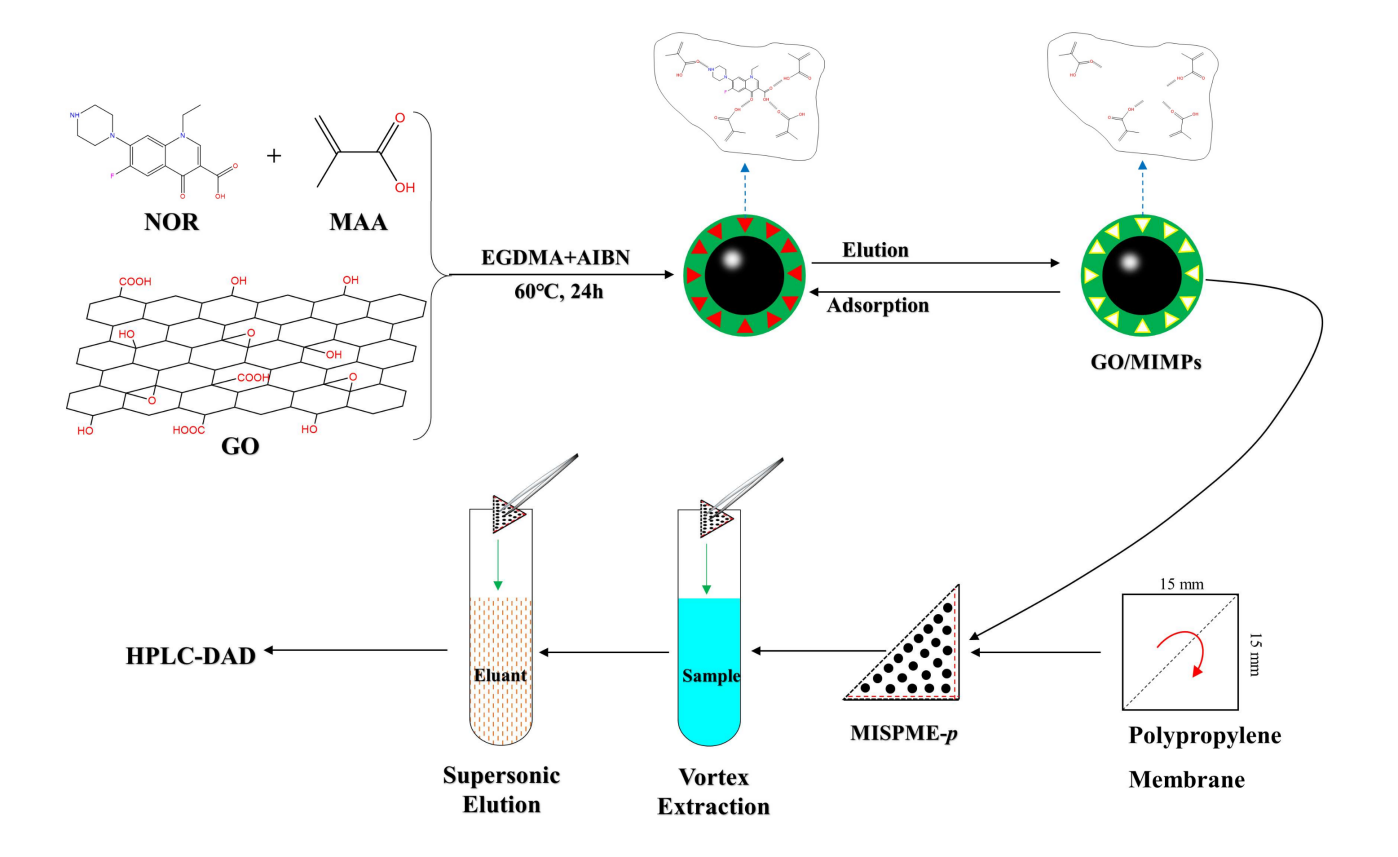
The preparation route of GO/MIPs and SPME procedure.

The MISPME procedure was established and is shown in Scheme 1. The polypropylene membrane (0.45 μm pore diameter) was cut to a square shape (15 mm × 15 mm), then folded into an equilateral triangle shape. One edge of the folder was heat-sealed by an impulse sealer. After the folder was washed in methanol for about 10 min by ultrasonic elution, 5 mg of GO/MIPs was added to the triangle folder, then the other edge was also heat-sealed and the molecularly imprinted solid-phase microextraction package (MISPME-*p*) was set. The obtained MISPME-*p* was stored in methanol and the MISPME procedure was performed as follows: A quantity of 10 mL of spiked sample was added in a 20 mL sample bottle, then 1.5 mL of acetonitrile was added, and pH was adjusted to 6 using HCl (1 mM) and NaOH (1 mM). The solution was sonicated for 2–3 min to make the solution homogeneous. Then the prepared MISPME-*p* was added to the sample solution. After vortex stirring for 5 min, the MISPME-*p* was transferred to another bottle with 1.5 mL of acetonitrile/acetic acid (70/30, *v/v*), and ultrasonicated for 5 min to desorb the norfloxacin. A quantity of 0.5 mL of eluent was vaporized with nitrogen and reconstituted with 0.5 mL of acetonitrile/water (19/81, *v/v*) for further analysis by HPLC-DAD.

### 2.8. Samples Preparation

The seawater was collected from Jiaozhou bay and the Shazikou aquaculture zone (Qingdao, China), and filtered through a 0.22 μm membrane to produce the water samples. The *Lateolabrax japonicus* were purchased from the local market, and the muscle tissues were cut into pieces and homogenized. A quantity of 1 g of muscle tissues and 10 mL of norfloxacin standard solution (0–10 μg L^−1^) was added to the centrifuge tube to mix completely. A quantity of 5 mL of acetonitrile/acetic acid (95/5, *v/v*) was then added to the tube as the extraction solvent. After ultrasonication for 15 min, the tube was centrifuged at 4000 rpm for 10 min and the supernatants were collected [20,33]. The extraction procedure was performed three times, and the supernatants were mixed and vaporized with nitrogen, and then reconstituted with 10 mL of water for analysis.

## 3. Results and Discussion

### 3.1. Characteristics of the Polymers

The GO/MIPs were prepared by precipitation polymerization (Scheme S1). In this route, GO was co-polymerized with EGDMA by the covalent reaction of the protic functional groups (e.g., hydroxyl, carboxyl) [43]. Then the pre-polymerization system of norfloxacin and MAA could be attached to the GO complex by non-covalent bonding through oxygen-containing groups under the action of EGDMA and AIBN. The molecularly imprinted microspheres were formed by heat initiation. After washing the template norfloxacin from the polymers, the imprinted holes with the same shape and complementary binding sites would be left in GO/MIPs [37]. When encountering norfloxacin again, the imprinted polymers would simulate the “antigen-antibody” molecular recognition mechanism and adsorb norfloxacin to the imprinted caves, thus completing the process of selective adsorption of norfloxacin.

**Figure 1:**
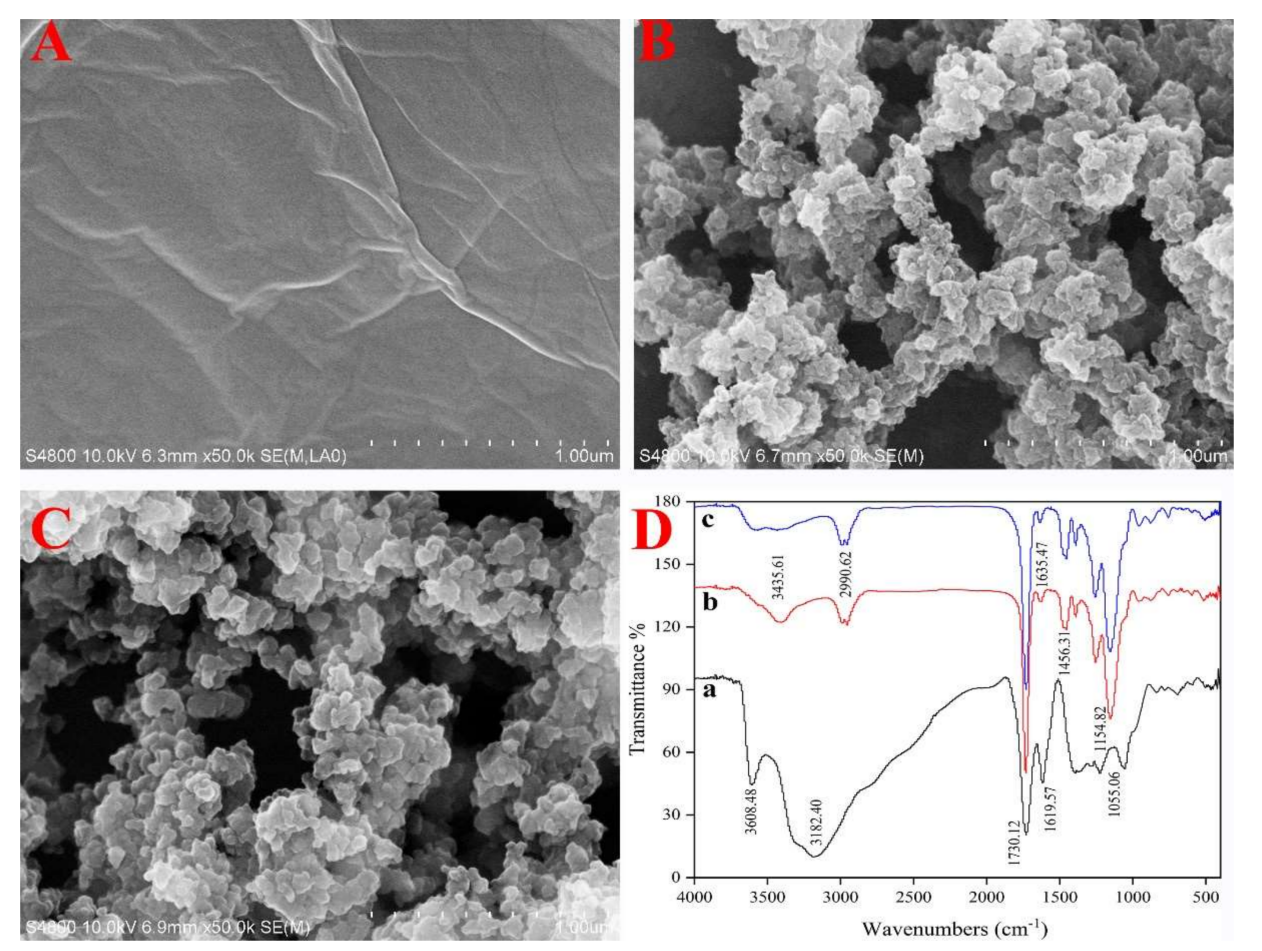
SEM images and FT−IR spectra of the polymers (**A**,a), GO; (**B**,b), GO/NIPs; (**C**,c), GO/MIPs; (**D**), FT−IR spectra.

To understand the characteristics of the morphology, structure, and functional groups of the synthesized polymers, SEM and FT-IR spectra of the polymers were studied and are shown in Figure 1. The obtained GO was layered with many folds on the surface (Figure 1A). The GO/NIPs and GO/MIPs (Figure 1B,C) were spherical. The BET specific surface area and pore size distribution of the polymers were measured and are shown in Figure S1. The results showed that the specific surface area of GO/MIPs (98.10 m^2^ g^−1^) was greater than GO/NIPs (67.75 m^2^ g^−1^). The BJL pore volume and the average size of the GO/MIPs were 0.18 cm^3^ g^−1^ and 102.62 Å, respectively, while those of GO/NIPs were 0.11 cm^3^ g^−1^ and 91.17 Å. The results indicated that GO/MIPs had a more porous structure than GO/NIPs caused by the template participation, which would provide a better mass transfer rate and adsorption capacity for the former.

FT-IR spectra (Figure 1D) reflected the functional groups in the polymers. The characteristic peaks at 3182 cm^−1^ and 2990 cm^−1^ represent the stretching vibration of O–H, and the characteristic peak near 1730 cm^−1^ represents the C=O group in carboxylic acid, which revealed there were oxygen-containing functional groups in GO. The feature peaks at 1154 cm^−1^ and 1260 cm^−1^ resulting from C–O vibrations (a,b in Figure 1D) in carboxylic acids and esters came from the involvement of MAA and EGDMA in the synthesis process which indicated the successful synthesis of the polymers [41]. Furthermore, the absorption peaks of GO/NIPs (b in Figure 1D) and GO/MIPs (c in Figure 1D) particles were similar, which meant that no templates were retained on the GO/MIPs.

### 3.2. Adsorption Capacity of GO/MIPs

**Figure 2:**
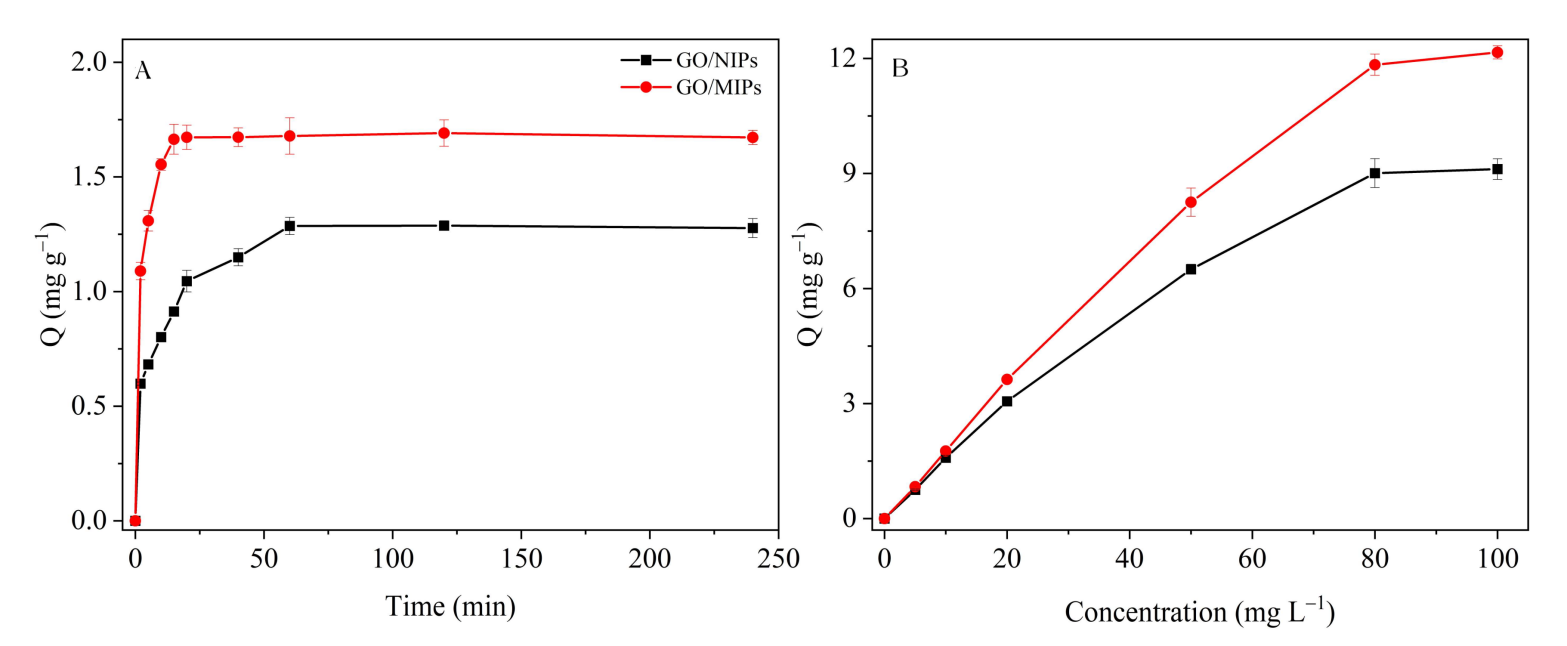
Dynamic (**A**) and static (**B**) adsorption of NOR by GO/MIPs and GO/NIPs.

To investigate the adsorption capacity of GO/MIPs, the adsorption rate of GO/NIPs and GO/MIPs to norfloxacin was researched by dynamic adsorption experiment (Figure 2A). The adsorption capacity of the two polymers to norfloxacin increased with time. GO/MIPs showed a faster adsorption rate for norfloxacin, reaching the adsorption equilibrium at about 20 min, while GO/NIPs reached the adsorption equilibrium at 60 min. The adsorption kinetics of the synthesized polymers were further investigated using the pseudo-first-order and pseudo-second-order equations by the following equations [44].

$$\mathrm{Pseudo}\text{-}\mathrm{first}\text{-}\mathrm{order}:\;\ln(Q_{e}-Q_{t})=\ln Q_{t}-K_{1}t$$

$$\mathrm{Pseudo}\text{-}\mathrm{second}\;\mathrm{order}:\;\frac{t}{Q_{t}}=\frac{1}{K_{2}Q_{e}^{2}}+\frac{t}{Q_{e}}$$

where Q_e_ (mg g^−1^) and Q_t_ (mg g^−1^) represent the amounts of norfloxacin adsorbed at equilibrium and time t, respectively, and K_1_ (min^−1^) and K_2_ (g mg^−1^ min^−1^) are the rate constants of sorption. Figure S2 showed the result of the fitted pseudo-first-order and pseudo-second order dynamics curve. The correlation coefficient (R^2^) values of the pseudo-second-order model for GO/MIPs (0.990) and GO/NIPs (0.952) were higher than those of the pseudo-first-order model (0.969, 0.879). Therefore, the pseudo-second-order equation of solute chemisorption on adsorbent was more suitable to describe the adsorption of norfloxacin into the polymers.

The static adsorption experiment showed that the adsorption capacity of GO/MIPs and GO/NIPs increased with the concentration of norfloxacin standard solution. The adsorption capacity of GO/MIPs was significantly higher than that of GO/NIPs (Figure 2B). The adsorption data were investigated using the Langmuir and Freundlich isothermal models by the following equations [44].

$$\mathrm{Langmuir}:\;\frac{C_{e}}{Q_{e}}=\frac{C_{e}}{Q_{m}}+\frac{1}{bQ_{m}}$$

$$\mathrm{Freundlich}:\;\ln Q_{e}=\ln K_{f}+\frac{1}{n}\ln C_{e}$$

where C_e_ (mg L^−1^) is the equilibrium concentration of norfloxacin in the solution, Q_e_ (mg g^−1^) and Q_m_ (mg g^−1^) are the amounts of norfloxacin adsorbed at equilibrium and maximum, respectively, K_f_ (mg g^−1^), n and b are the constants of Langmuir and Freundlich. The Langmuir model assumes that the adsorbent has a uniform surface and has similar and energy-equivalent binding sites, which can provide monolayer adsorption. The Freundlich model is usually used to describe multilayer adsorption on heterogeneous surfaces. The obtained results for the Langmuir and Freundlich models are shown in Figure S3; the correlation coefficient R^2^ was evaluated to judge the applicability of the models. The R^2^ values of GO/MIPs (0.988) and GO/NIPs (0.976) in the Langmuir model were both greater than those in the Freundlich model (0.889, 0.913). Therefore, the adsorption experiment data of norfloxacin on GO/MIP fitted the Langmuir isotherm model more closely, indicating that the synthesized imprinted polymers had a uniform surface and regular monolayer adsorption to norfloxacin.

In addition, the pore sizes of GO/MIPs were larger than that of GO/NIPs which could assist in improving the mass transfer rate and adsorption capacity. Specific binding sites in GO/MIPs could bind to norfloxacin through non-covalent bonding, which also improved the identification and binding capacity to NOR. Meanwhile, the selectivity of GO/MIPs was investigated with two structural analogs of norfloxacin and two other matrix compounds (Supplementary Materials, Figure S4). The GO/MIPs had higher selective adsorption capacity for norfloxacin than the other compounds. The GO/MIPs exhibited more significant adsorption selectivity for quinolone antibiotics than the sulfa antibiotics, which was mainly due to the effective imprinting effects and differences in size, functional group type, and the location of analytes. Therefore, comparing the adsorption differences between GO/MIPs and GO/NIPs, it was found that the former had selective adsorption for norfloxacin but only non-specific adsorption for sulfadiazine and sulfadiazine.

### 3.3. Optimization of the MISPME Procedure

#### 3.3.1. Type and Volume of Organic Cosolvent

**Figure 3:**
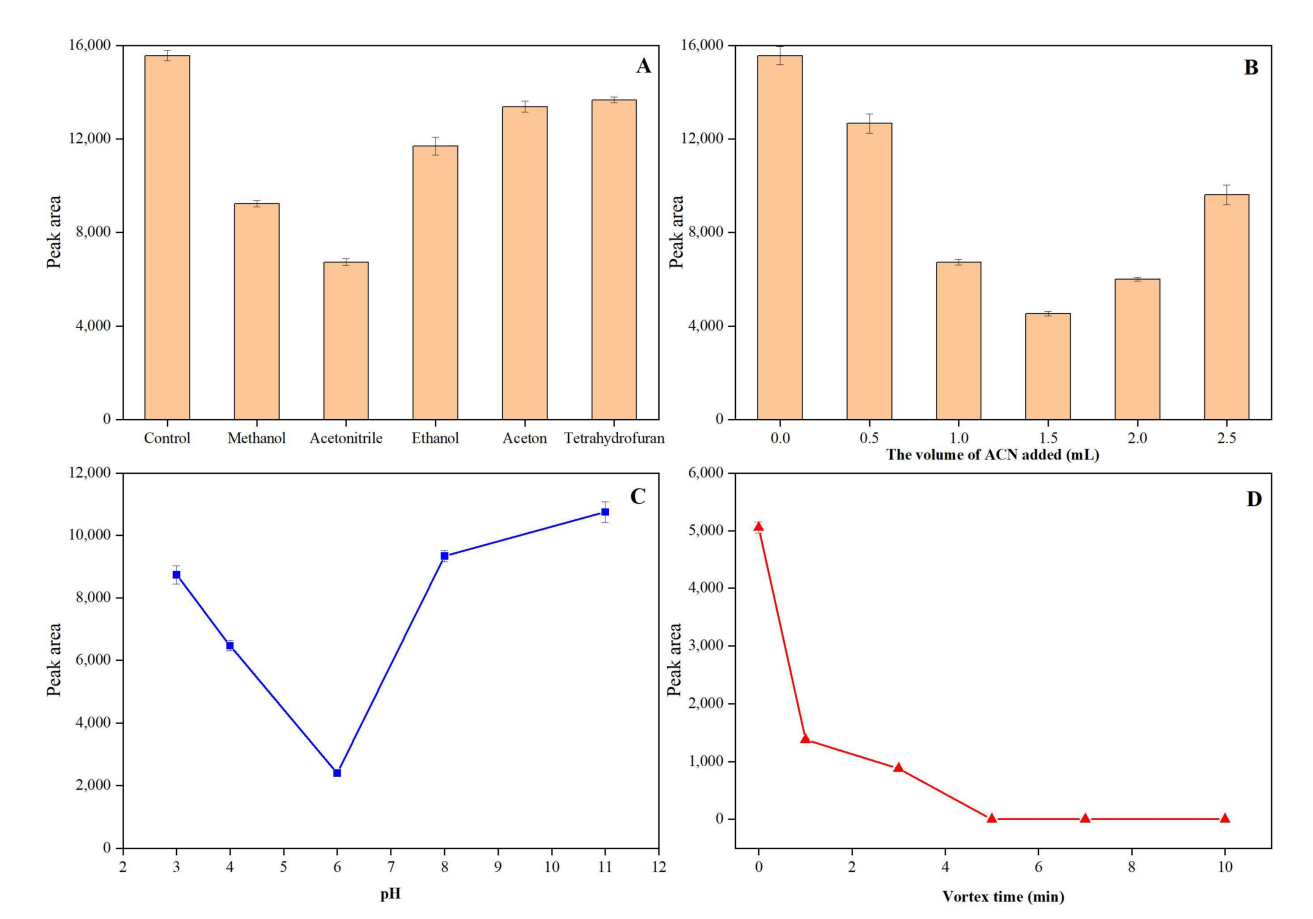
Effects of different types (**A**) and volumes (**B**) of organic cosolvent, pH (**C**) and vortex time (**D**) on extraction efficiency.

The composition of organic solvent and water will affect the distribution of norfloxacin in organic phase and aqueous phase, and indirectly affect the adsorption of norfloxacin by polymers [45]. The effect of polymers on norfloxacin adsorption was investigated when different types and volumes of organic solvents were added to the solution. Five organic cosolvents (methanol, acetonitrile, ethanol, acetone, and tetrahydrofuran) were selected and added to 10 mL of norfloxacin standard solution, then MISPME-*p* was added to the solution. After vortex extraction for 10 min, the amount of norfloxacin remaining in the solution was measured, which was represented in the characteristic chromatographic peak (Figure 3A). The addition of organic solvent promoted the adsorption of norfloxacin by MISPME-*p* because the organic solvent could change the dispersion of norfloxacin in solution. In addition, the solution containing acetonitrile had the least residual norfloxacin, indicating that acetonitrile could greatly promote the adsorption capacity of MISPME-*p* for NOR in the solution. The polarity of the organic solvent was in the order acetonitrile > methanol > EtOH > acetone > tetrahydrofuran, which indicated the highly polar aprotic organic solvent could promote the adsorption of polymers to norfloxacin. Therefore, the effect of the acetonitrile additive volume on the adsorption property of MISPME–*p* was further studied. It was observed that 1.5 mL of acetonitrile gave the best extraction efficiency (Figure 3B). Thus, 1.5 mL of acetonitrile was selected as the best organic cosolvent for subsequent research.

#### 3.3.2. Effect of pH and Extraction Time

The pH of the sample solution seriously affected the binding efficiency between the adsorbent and the analyte. The pH determined the existing form of norfloxacin for containing acidic and basic functional groups in solution (such as cation, anion, molecule, etc.), which further influenced the binding ability between the compound and the adsorbent [46]. The extraction efficiency of GO/MIPs for NOR in the solution of different pH (3–11) was investigated; the result is shown in Figure 3C. The GO/MIPs had high adsorption capacity for norfloxacin at pH 6, which was mainly attributed to the present form of norfloxacin in solution being slightly positively charged with pKa_1_ and pKa_2_ of 6.34 and 8.75, respectively, enabling combination with the binding sites in the GO/MIPs through hydrogen bonds. When pH is too high or too low, the adsorption of norfloxacin by the polymers will decrease. Therefore, pH 6 was chosen for the subsequent experiment. The extraction time of the vortex was also optimized to maximize the extraction efficiency of polymers to norfloxacin (Figure 3D). When vortexed for 5 min, norfloxacin was fully combined with GO/MIPs. Therefore, 5 min was selected as the optimal extraction time.

#### 3.3.3. Effect of Eluent Type and Volume

**Figure 4:**
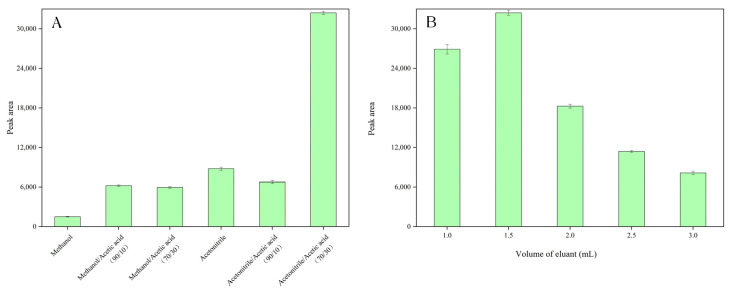
Effects of different types (**A**) and volumes (**B**) of eluents on elution efficiency.

Elution is the process of releasing the target component from the adsorbent. The desorption efficiency of the analyte needs to use the appropriate desorption solvent during SPME. Different proportions of methanol/acetic acid and acetonitrile/acetic acid were investigated as the eluant. The elution procedure was performed under ultrasound. After the MISPME-*p* was vortexed for extraction of the norfloxacin, the MISPME-*p* was immersed in the eluent and subject to ultrasound for 5 min to elute the adsorbed norfloxacin; the result is shown in Figure 4A. After adding some acetic acid to the methanol, the eluent could improve the desorption efficiency of norfloxacin [47]. The elution efficiency of acetonitrile/acetic acid (90/10, *v/v*) on norfloxacin was not significantly improved compared with that of acetonitrile alone; however, acetonitrile/acetic acid (70/30, *v/v*) solution as the eluent had better desorption efficiency for norfloxacin. Therefore acetonitrile/acetic acid (70/30, *v/v*) was selected as the eluent to further investigate the effect of volume on the elution efficiency. When the volume was 1.5 mL, the elution efficiency for norfloxacin was optimal (Figure 4B). When the volume was low, the solution was not sufficient to immerse the MISPME-*p* and could not elute the norfloxacin to the maximum. When the volume was large, the eluent efficiency of norfloxacin was reduced due to the dilution effect. Therefore, the optimal volume of eluent was 1.5 mL.

The final optimum extraction conditions were as follows: organic cosolvent, 1.5 mL of acetonitrile; pH 6; vortex extraction time, 5 min; and elution solution, 1.5 mL of acetonitrile/acetic acid (70/30, *v/v*). The enrichment factor (EF) of the MISPME procedure was 7.01 with the above conditions, as calculated with the following equation.

EF = C_f_/C_0_

where C_f_ and C_0_ are the final concentrations of norfloxacin found in the desorption solvent and the original sample solution, respectively.

### 3.4. Analytical Performance for NOR in Real Samples

To verify the performance of the established MISPME method, seawater and fish samples collected from the Jiaozhou bay and the Shazikou breeding area were analyzed. A quantity of 10 mL of sample solution was processed under the optimal MISPME conditions and determined by HPLC–DAD. The results are shown in Table S1. After MISPME treatment, the standard recovery rate of seawater and fish samples was above 90%, and a trace amount of NOR was detected in the seawater in the Shazikou area, while no norfloxacin was detected in the seawater of the Jiaozhou bay and fish samples.

The detection limit (LOD) and quantitation limit (LOQ) of norfloxacin were calculated based on a signal-to-noise ratio of 3 and 10, which was 0.15 μg L^−1^ and 0.50 μg L^−1^ for the water samples, and 0.10 μg kg^−1^ and 0.33 μg kg^−1^ for the fish samples, respectively. In addition, a comparison of the methods for norfloxacin determination is presented in Table 1, which shows that the proposed MISPME procedure was effective and simple with satisfactory recovery and could provide a new research approach for norfloxacin detection for the marine environment.

### 3.5. Reusability

An experiment was carried out to study the recyclability of MISPME-*p*. After each MISPME procedure, MISPME-*p* was ultrasonic cleaned in 2 mL of acetonitrile/acetic acid (70/30, *v/v*) for 5 min repeated four times, followed by vortex cleaning in 5 mL of methanol for 2 min repeated three times to regenerate MISPME-*p*. The recovery for norfloxacin was stably above 86% after using eight times with regenerated MISPME-*p*, as shown in Figure S5. The results indicated that the MISPME-*p* had satisfactory reusability and that the reliability of the method was assured.

## 4. Conclusions

In conclusion, a novel MISPME procedure was proposed for the determination of norfloxacin combined with HPLC–DAD for marine environment samples with GO/MIPs as adsorbents of MISPME-*p*. The GO/MIPs were synthesized using MAA as a functional monomer, EGDMA as a crosslinking agent and AIBN as an initiator along with GO. Morphological analysis revealed the GO/MIPs had a loose porous structure and uniform surface. That the polymers showed regular monolayer adsorption to norfloxacin was demonstrated by application of the Langmuir isotherm model. The pH, type and volume of the organic solvent, adsorption time, type and volume of eluent were optimized for the MISPE procedure. The newly established method showed high recovery and satisfactory LOD for the determination of norfloxacin, which was successfully applied in fish and seawater sample determination. Trace norfloxacin was detected from seawater in the Shazikou aquaculture zone. The proposed MISPME-*p* showed satisfied efficiency and reusability, which supported its potential for analyzing trace antibiotics in the marine environment.

## Figures and Tables

**Table 1:** Comparison of the methods for the determination of norfloxacin.

Sample	Extraction Method	Detection	LOD	Recovery	Reference
Chicken	^a^ MISPE	HPLC–FLD	6.00 μg/kg	94.0–101.0%	[1]
Water	^b^ MIP-MEPS	LC–MS/MS	3.80 μg/L	82.0–104.0%	[3]
Fish	^c^ DMI-MSPD	HPLC–FLD	0.14 μg/kg	95.0–99.2%	[48]
SeawaterFish	MISPME	HPLC–DAD	0.15 μg/L0.10 μg/kg	90.1–102.7%	This work

^a^: Molecularly imprinted solid-phase extraction; ^b^: Molecularly imprinted polymer microextraction by packed sorbent; ^c^: Dummy molecularly imprinted matrix solid-phase dispersion.

## Data Availability

Not applicable.

## Supplementary Materials

The following supporting information can be downloaded at: https://www.mdpi.com/article/10.3390/polym14091839/s1, Figure S1: Nitrogen adsorption-desorption plots and BJH pore size distribution for the polymers (a/b, GO/MIPs; c/d, GO/NIPs); Figure S2: The fitting curves of adsorption kinetics with the pseudo-first-order and pseudo-second-order models; Figure S3: Diagram of the Langmuir and Freundlich isotherms; Figure S4: The selective adsorption capacity of the polymers for NOR and competing compounds; Figure S5: The reusability of MISPME-*p*. Table S1: Recovery of the spike samples after MISPME procedure (n = 3); Scheme S1: Illustration of the preparation of GO/MIPs composites.
