# Supplementary material for: Graphene Oxide Molecularly Imprinted Polymers as Novel Adsorbents for Solid-Phase Microextraction for Selective Determination of Norfloxacin in the Marine Environment
Source: Polymers (Basel). 2022 Apr 29;14(9):1839. doi: 10.3390/polym14091839 (PMC9101591; doi:10.3390/polym14091839)
Supplement: Supplementary file 1 [file polymers-14-01839-s001.zip › polymers-1694081-supplementary.pdf]

Graphene oxide molecularly imprinted polymers as novel adsorbents for solid-phase microextraction for selective determination of norfloxacin in the marine environment.

Jianlei Chen[1,2], Liju Tan[2], Zhengguo Cui[1,*], Keming Qu[1] and Jiangtao Wang[2,*]

S1. Selectivity of the polymers

The structural analog ofloxacin (OFL), ciprofloxacin (CIP) and other matrix compounds, sulfadiazine (SDZ) and sulfamethazine (SMZ), were chosen to evaluate the selectivity of GO/MIPs and GO/NIPs (Fig S1). A quantity of 5 mg of MIP@GO or NIP@GO were added into 1 mL of the above compounds' standard solution (10 mg L^{-1}), respectively, and shaken for 2 h at room temperature. The mixture was passed through a 0.22 μm filter membrane and the NOR in the filtrate was determined by HPLC-DVD. The imprinting factor (IF) of each compound was determined by the following equation.

$$IF = Q_M / Q_N$$

where Q_M and Q_N (mg L^{-1}) represent the binding amounts of the test analytes on the MIP@GO and NIP@GO, respectively.

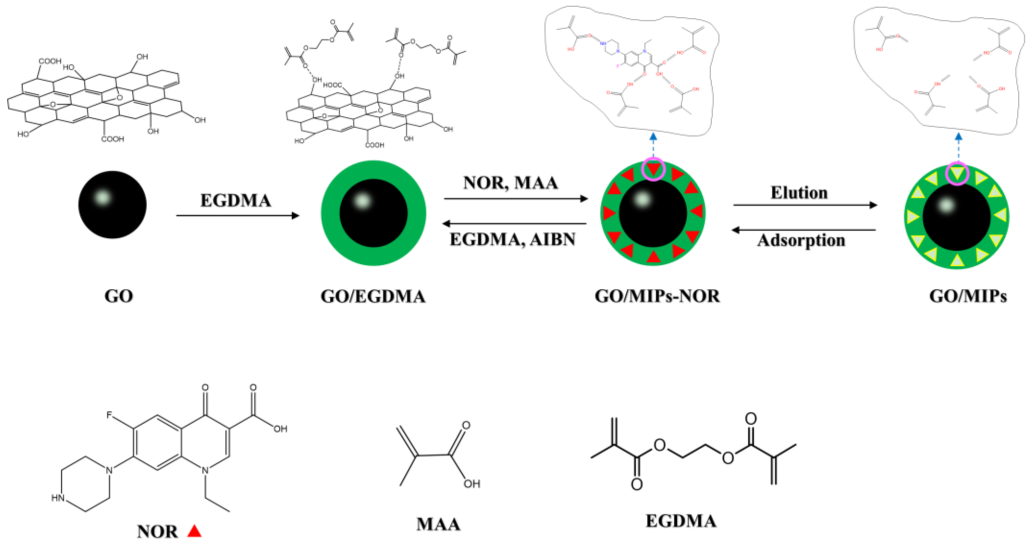

Scheme S1. Illustration of the preparation of GO/MIPs composites.

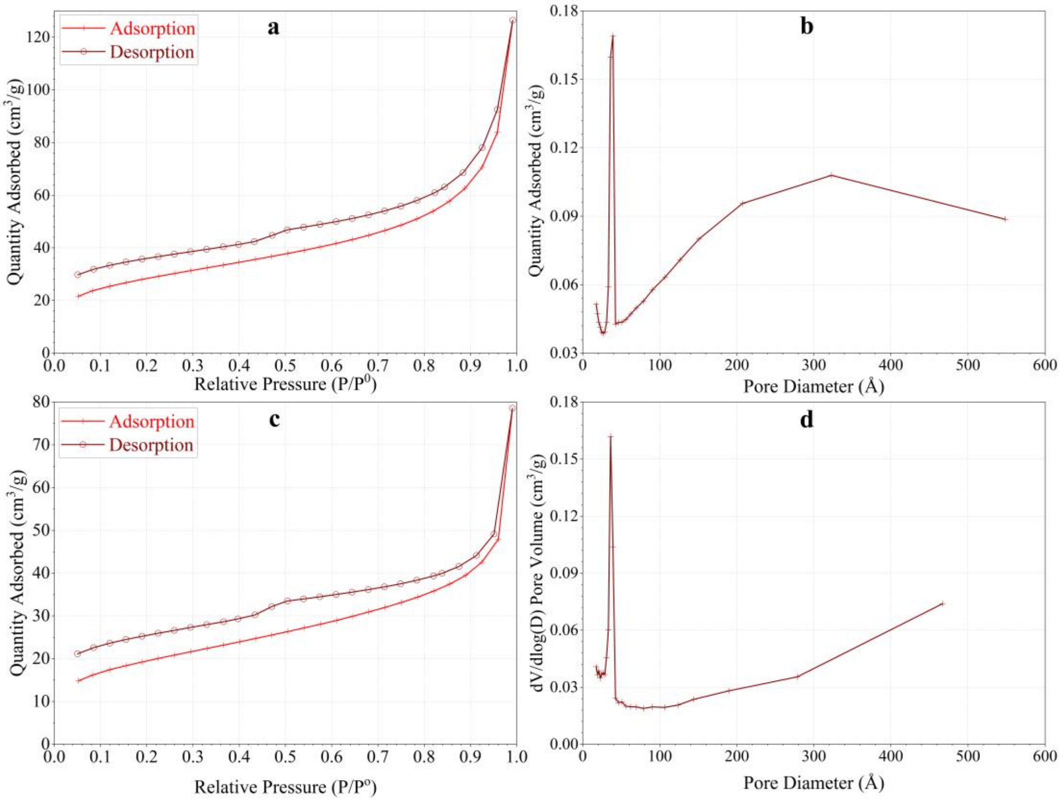

Figure S1. Nitrogen adsorption-desorption plots and BJH pore size distribution for the polymers (a/b, GO/MIPs; c/d, GO/NIPs).

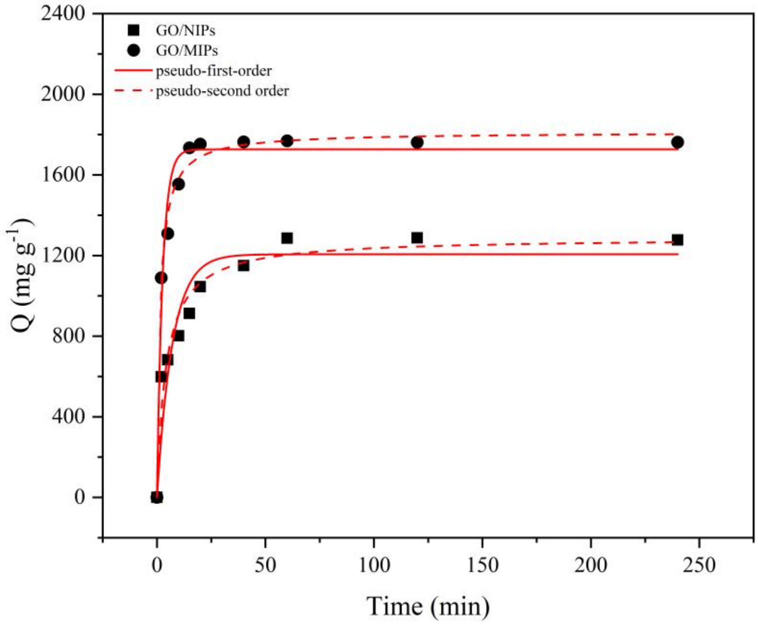

Figure S2. The fitting curves of adsorption kinetics with the pseudo-first-order and pseudo-second-order models.

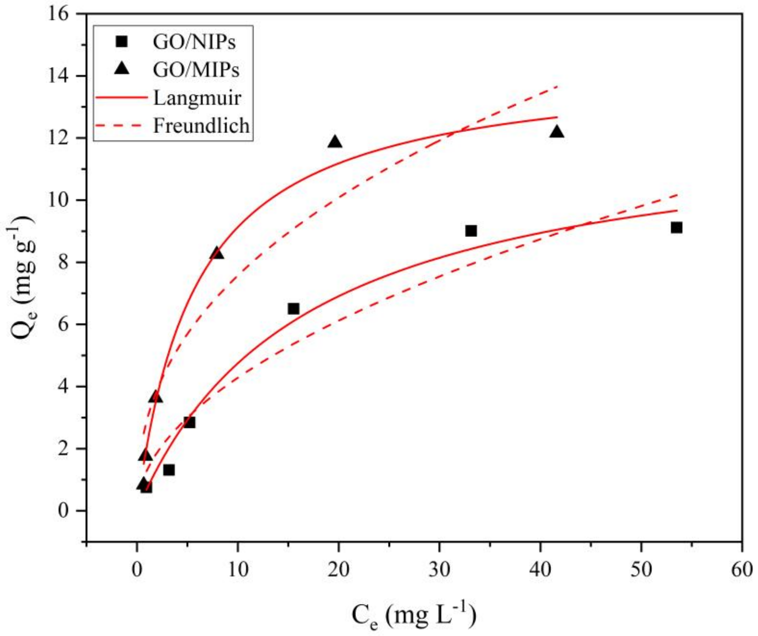

Figure S3. Diagram of the Langmuir and Freundlich isotherms.

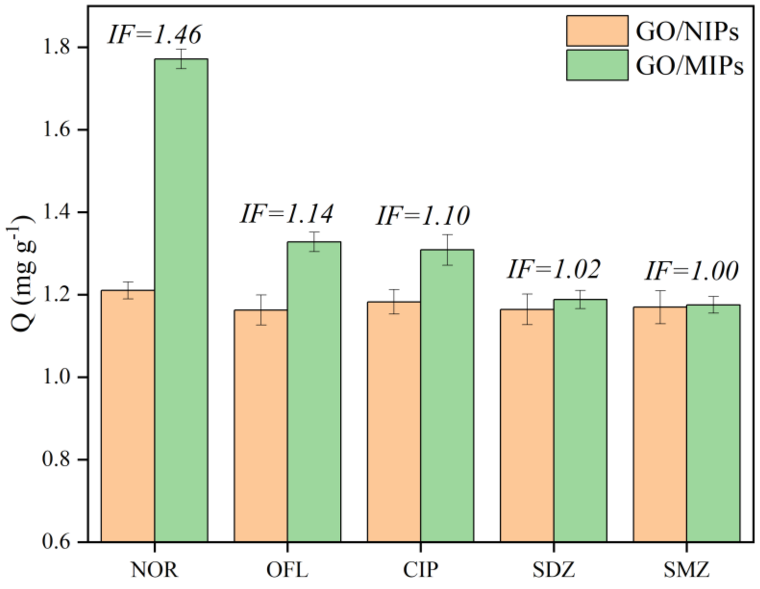

Figure S4. The selective adsorption capacity of the polymers for NOR and competing compounds.

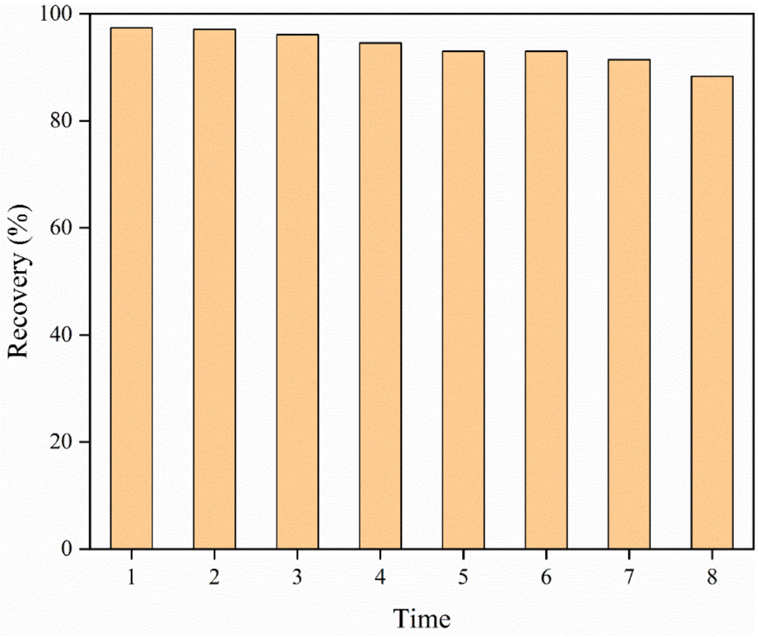

Figure S5. The reusability of MISPME-*p*.

Table S1. Recovery of the spike samples after MISPME procedure (n=3).

**Samples**	**Spiked (μg L-1)**	**Elution (μg L-1)**	**Recovery (%)**	**RSD (%)**	**Detected concentration (μg L-1)**
Seawater (Jiaozhou Bay)	0	0			ND
	2	13.43	95.78	2.78	
	4	27.54	98.23	5.29	
	8	52.80	94.15	3.14	
	10	63.39	90.44	2.60	
Seawater (Shazikou wharf)	0	4.70		2.15	0.67
	2	19.10	102.73	4.81	
	4	32.59	99.48	2.06	
	8	58.63	96.16	5.11	
	10	75.16	100.52	4.27	
Lateolabrax japonicus (Jiaozhou Bay)	0	0			ND
	2	12.98	92.55	2.36	
	4	25.27	90.13	4.91	
	8	52.56	93.72	4.02	
	10	64.08	91.41	3.32	
Lateolabrax japonicus (Shazikou wharf)	0	0			ND
	2	12.74	90.86	4.65	
	4	26.46	94.37	5.07	
	8	54.19	96.63	4.14	
	10	64.42	91.90	2.28	

ND=not detected.
